# Generation and Characterization of Novel iPSC Lines from a Portuguese Family Bearing Heterozygous and Homozygous *GRN* Mutations

**DOI:** 10.3390/biomedicines10081905

**Published:** 2022-08-06

**Authors:** Ana Rafaela Oliveira, Solange Martins, Giuseppe Cammarata, Mariana Martins, Ana Maria Cardoso, Maria Rosário Almeida, Maria do Carmo Macário, Isabel Santana, João Peça, Ana Luísa Cardoso

**Affiliations:** 1 CNC—Center for Neuroscience and Cell Biology, University of Coimbra, 3004-504 Coimbra, Portugal; 2 Center for Innovative Biomedicine and Biotechnology (CIBB), University of Coimbra, 3004-504 Coimbra, Portugal; 3 MIT-Portugal Ph.D. Program, NOVA School of Science and Technology, New University of Lisbon, 2829-516 Caparica, Portugal; 4 Institute for Interdisciplinary Research (IIIUC), University of Coimbra, 3030-789 Coimbra, Portugal; 5 Doctoral Programme in Experimental Biology and Biomedicine (PDBEB), CNC—Center for Neuroscience and Cell Biology, University of Coimbra, 3004-504 Coimbra, Portugal; 6 Faculty of Pharmacy, University of Coimbra, 3000-548 Coimbra, Portugal; 7 Neurology Department, Centro Hospitalar e Universitário de Coimbra, 3004-531 Coimbra, Portugal; 8 Faculty of Medicine, University of Coimbra, 3004-504 Coimbra, Portugal; 9 Department of Life Sciences, Faculty of Science and Technology, University of Coimbra, 3000-456 Coimbra, Portugal

**Keywords:** *GRN* mutations, Portuguese family, frontotemporal lobar degeneration, reprograming, human-induced pluripotent stem cells

## Abstract

Mutations in granulin (*GRN*) have been associated with neurodegenerative diseases, such as frontotemporal lobar degeneration (FTLD) and neuronal ceroid lipofuscinosis (NCL). In Portugal, *GRN* mutations account for around half of all FTLD cases with known genetic origin. Here, we describe the generation and characterization of three human-induced pluripotent stem cell (hiPSC) lines from a Portuguese family harboring heterozygous and homozygous *GRN* mutation. hiPSCs were reprogrammed from human dermal fibroblasts by episomal nucleofection of the Yamanaka factors. The new generated lines were positive for pluripotency markers, could be further differentiated to cells expressing all trilineage markers, and presented a normal karyotype. They were also capable of differentiating into 3D brain organoids and presented a significant decrease in progranulin protein levels. Hence, these cell lines constitute suitable new tools to elucidate the pathophysiological mechanisms associated with the *GRN* mutations in the context of FTLD.

## 1. Introduction

Frontotemporal lobar degeneration (FTLD) is the second most common form of dementia in people aged <65 years old and comprises an heterogenous group of highly hereditable and rapidly progressing neurodegenerative diseases, mostly characterized by behavioral, language and motor impairments. In 2006, heterozygous mutations in *GRN* were identified as a cause of familial FTLD presenting with inclusions of the TAR-DNA binding protein 43 (TDP-43) [1,2]. Mutations in *GRN*, together with mutations in microtubule-associated protein tau (*MAPT*) or expansion of the chromosome 9 open reading frame 72 (*C9orf72*), account for 5% to 10% of all FTLD cases [3,4]. In the specific case of the Portuguese population, *GRN* mutations are relatively frequent and account for around half of all genetic FTLD diagnoses [4], showing almost 100% penetrance by 80 years of age.

Progranulin, the protein encoded by the *GRN* gene, is a conserved 593 amino acid and 88-kDa protein, which includes 7.5 cysteine-rich granulin domains. In the brain, progranulin is expressed both by immune cells and by subsets of neurons and has been implicated in multiple functions required for neuronal survival [1,2], including axonal growth, synaptogenesis [5,6] and neuroinflammation [7,8,9,10]. In addition to its extracellular functions, mainly mediated through interaction with the tyrosine kinase ephrin type-A receptor 2 (EphA2) and the Notch signaling pathway [11], progranulin (PGRN) has also been suggested to play an important role in autophagy and lysosomal regulation, acting as a chaperone for lysosomal enzymes involved in protein degradation [12].

**Figure 1:**
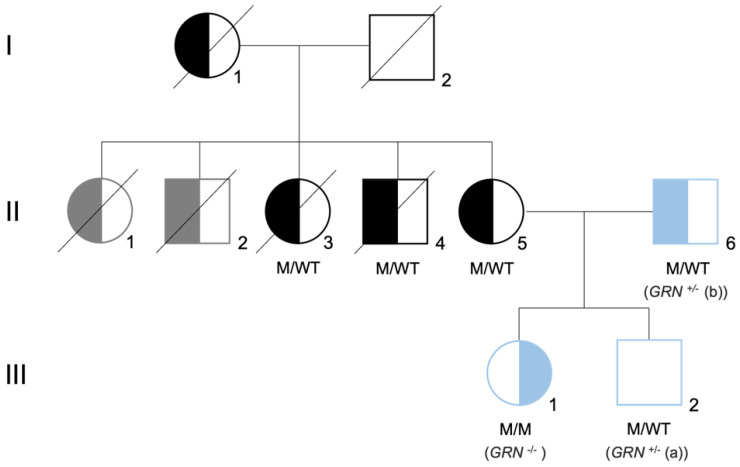
Family tree of the Portuguese family carrier of the *GRN* mutation. Family tree representative of the mutation status for *GRN* mutation c.900_901dupGT. Both parents (II5 and II6) are heterozygous for the mutation (M/WT), presenting with the behavioral variant of FTLD. The daughter (II1) is homozygous for the mutation (M/M), presenting with neuronal ceroid lipofuscinosis (CLN11). The son (II2) is heterozygous for the mutation (M/WT) and did not present symptoms at the time of the skin biopsy. Left-side filled black/blue symbols represent individuals diagnosed with behavioral variant of FTLD, while right-side filled black/blue symbols represent individuals diagnosed with neuronal ceroid lipofuscinosis. Left-side filled gray symbols represent signs of dementia plus Parkinsonism and the white square represents asymptomatic individuals. The generation is indicated by Roman numerals on the left side of the tree. The samples used in this study are represented in blue and are referred as *GRN*^+/^^−^ (a), *GRN*^+/^^−^ (b), and *GRN*^−/^^−^. Image adapted from [18,19].

In addition to FTLD, deficiencies in the *GRN* gene can cause other neurodegenerative conditions, in an allele dose-dependent manner. Clinical symptoms of patients with *GRN* mutations, including the age of disease onset, are extremely variable, even within same-family carriers of an identical pathogenic mutation [13,14]. Interestingly, homozygous mutations in *GRN* were not initially observed, leading to the hypothesis that the loss of *GRN* in both alleles might cause embryonic death [15]. This changed in 2012, when a complete deficiency of progranulin, caused by a homozygous *GRN* loss-of-function mutation, was reported in two siblings diagnosed with adult-onset neuronal ceroid lipofuscinosis (NCL) type 11, presenting with visual loss, dementia and epilepsy [16,17]. In 2016, a c.900_901dupGT mutation in exon 9 of the *GRN* gene (p.Ser301Cysfs*61) was identified in a young Portuguese female, originating the third case of NCL due to a homozygous *GRN* mutation [18], and the first one segregating in a family with confirmed pathology of FTLD [19] (Figure 1).

The association of progranulin with a late-onset type NCL further strengthens the hypothesis of a relevant role for this protein both in lysosomal homeostasis and lipid metabolism [16,20]. This was further confirmed by several studies in *GRN^−/−^* mice, which showed an abnormal accumulation of lipofuscin deposits in the brain [4,21], in addition to changes in lysosome size and activity, reduced activity of autophagy mediators [22] and an increase in microglia-dependent complement activation that led to abnormal synapse elimination and neuronal loss [10].

In addition to rare loss of function *GRN* mutations, single-nucleotide polymorphisms (SNPs) in the *GRN* locus, such as rs5848, rs2269906 and rs850738, have been identified by genome-wide association studies as genetic determinants of other neurologic diseases, including Alzheimer’s disease, limbic-predominant age-related TDP-43 encephalopathy (LATE), amyotrophic lateral-sclerosis (ALS) and FTLD caused by expansion of *C9orf72* and Gaucher disease [23]. All these SNPs have been related to decreased levels of circulating progranulin, which have also been reported in idiopathic Parkinson disease and autism [24,25]. This commonality in a risk gene for neurodegeneration is almost unheard of and suggests that progranulin is a critical regulator of brain health, constituting a uniquely attractive therapeutic target [23].

Despite the undeniable link between pathogenic mutations in *GRN* and both FTLD and NCL, the underlying molecular and cellular pathways that explain the contribution of progranulin to the pathophysiology of these diseases, as well as the reason for brain susceptibility to progranulin loss, remain to be fully elucidated. This is partly due to the poor face validity of *GRN^+/−^* mice, as well as the lack of robust human models.

Human-induced pluripotent stem cells (hiPSCs) offer remarkable opportunities to study the molecular mechanisms of this type of mutation, due to their ability to differentiate into various cell types [26], which enables the development and study of human neurons while preserving the genetic background of the individuals. In fact, several studies using hiPSCs from patients with *GRN* mutations have reported that iPSC-derived neurons display many of the disease phenotypes observed in FTLD patients [5,27,28,29,30,31,32,33]. Almeida et al. demonstrated a decrease in *GRN* mRNA and PGRN protein levels and an increase in cytoplasmic TDP-43 in iPSCs-derived neurons from an FTLD carrier of a heterozygous *GRN* nonsense mutation [27]. Additionally, compromised cortical neuronal differentiation was also observed in *GRN* iPSCs-derived neurons [32]. The lysosomal function of progranulin has also been the subject of studies employing iPSCs models, which have validated the involvement of cathepsins in the cleavage of progranulin in the lysosome [30,33]. Moreover, iPSC-derived neurons have been investigated as possible therapeutic strategies to modulate progranulin levels and rescue the FTLD phenotype [5,28,29,31], showing the potential of these cells to address both questions related with the pathophysiological mechanisms of the disease and with possible therapeutic venues, in a human context.

IPSCs are powerful tools not only because they are able to differentiate into different neuronal populations, but because they can originate other cell types, including immune cells such as microglia, which are showing increasing relevance in *GRN*-associated diseases [10,34,35,36]. More recently, iPSCs have also been used for the generation of even more promising and complex models, such as brain organoids, which can be valuable tools to model the different genetic causes of FTLD [37,38].

Here, we describe the generation and characterization of new FTLD and NCL patient-specific iPSC lines, bearing the c.900_901dupGT *GRN* mutation, which we further employed to generate a human 3D model of *GRN* haploinsufficiency. We believe these lines constitute promising new tools that will allow future studies addressing the mechanistic aspects of progranulin function and other outstanding questions regarding the impact of progranulin deficiency in the context of aging and disease.

## 2. Materials and Methods

### 2.1. Fibroblasts Culture from Skin Biopsy

Skin punch biopsies from one healthy control (36 years of age, *GRN^+/+^*) and three individuals from the same family, harboring *GRN* mutations—one female (III1; 34 years of age, *GRN^−/−^*) and two males (III2 and II6; 29 years of age and 63 years of age, respectively, *GRN^+/−^*)—were collected at Centro Hospitalar e Universitário de Coimbra (CHUC), following approval by the Ethics Committee of the Faculty of Medicine, University of Coimbra (Project CE-028/2016 and Project OBS.SF.88-2021) and according to the principles for medical research involving human subjects stated in the Declaration of Helsinki. Informed consent was obtained from all donors. Data were treated confidentially according to the General Data Protection Regulation (GDPR), Regulation (EU) 2016/679 of the European Parliament and of the Council of 27 April 2016. The dissection of each skin punch biopsy and isolation of fibroblasts were performed following the protocol described by Vangipuram [39]. In brief, the epidermis was excluded, and the dermis was cut into small pieces and placed on a 6-well plate (Thermo Fisher Scientific, Waltham, MA, USA) (3 pieces/well). The samples were covered with a coverslip and fibroblast medium (DMEM—D5648 (Merk, Darmstadt, Germany), supplemented with 44 mM NaHCO_3_ (Thermo Fisher Scientific, Waltham, MA, USA), 1 mM Na-pyruvate (Gibco, Waltham, MA, USA), 1% penicillin/streptomycin (Gibco, Waltham, MA, USA) and 10% of heat-inactivated FBS (Gibco, Waltham, MA, USA), pH 7.3. The cells were maintained at 37 °C in a humidified atmosphere containing 5% CO_2_. Once fibroblasts emerged and became confluent, the cells were expanded and subsequently used for reprogramming.

### 2.2. Generation and Maintenance of Human-Induced Pluripotent Stem Cells (hiPSCs)

Human dermal fibroblasts were reprogrammed by episomal nucleofaction of the Yamanaka factors, as initially described by Howden et al., with several modifications [40]. Human dermal fibroblasts were harvested and 1 × 10^6^ cells per condition were resuspended in 100 μL PBS 1× The cell suspension and 1.5 μg each of pCXLE-hOCT3/4-shp53-F (Addgene, #27077), pCXLE-hUL (Addgene, #27080), pCXLE-hSK (Addgene, #27078) and pSimple-miR302/367 (Addgene, #98748) were added to a 0.2 cm cuvette (Mirus, Madison, WI, USA) and electroporated in an Amaxa Nucleofactor II (Lonza, Basel, Switzerland) device. Following electroporation, cells were plated on a 10 cm gelatin-coated tissue culture plate with fibroblast medium and incubated at 37 °C and 5% CO_2_. The medium was replaced every day with fresh medium containing 0.5 mM sodium butyrate (Stem Cell Technologies, Vancouver, BC, Canada). On day 7, cells were dissociated and plated on 6-well Matrigel-coated plates (Thermo Fisher Scientific, Waltham, MA, USA) with medium containing 0.5 mM sodium butyrate (Stem Cell Technologies, Vancouver, Canada). On day 8, the medium was replaced by mTeSR Plus (Stem Cell Technologies, Vancouver, Canada) with 0.5 mM sodium butyrate (Stem Cell Technologies, Vancouver, Canada) and changed every other day until day 12. From day 12, the medium was replaced every other day with mTeSR Plus (Stem Cell Technologies, Vancouver, Canada), without sodium butyrate, until hiPSC colonies emerged. Emerging colonies were manually collected for single cell clone expansion. After hiPSC isolation, cells were maintained and expanded in mTeSR Plus (Stem Cell Technologies, Vancouver, Canada) with manual passages until around passage 4. Thereafter, the ReLeSR (Stem Cell Technologies, Vancouver, Canada) passaging reagent was used every time cells reached 80% confluency. Full characterization of each hiPSC line was only performed after passage 15 and cells were used for brain organoid generation after passage 20.

### 2.3. Trilineage Assay: In Vitro Spontaneous Differentiation

hiPSC differentiation into the three germ layers was performed as previously described [41]. Briefly, hiPSCs from each cell line were harvested and 9000 cells per well were plated on a 96-well cell suspension plate (Greiner Bio-One, Kremsmünster, Austria) for embryoid body (EB) formation [42], using EB formation medium supplemented with 10 μM ROCK inhibitor Y-27632 from the STEMdiff Cerebral Organoid Kit (Stem Cell Technologies, Vancouver, Canada). Five days later, the EBs were plated on Matrigel-coated μ-slide 8-well ibiTreat chamber slides (Ibidi, Gräfelfing, Germany) in differentiation medium (DMEM/F-12 (Gibco, Waltham, MA, USA), 20% heat-inactivated FBS, 1% MEM non-essential amino acid solution (Gibco, Waltham, MA, USA), 0.1 mM β-mercaptoethanol (Merk, Darmstadt, Germany ) and 1% penicillin/streptomycin (Gibco, Waltham, MA, USA)). The medium was changed every other day until day 14 of differentiation. On day 14, the cells were fixed with 4% paraformaldehyde (PFA) for 20 min at room temperature (RT) and kept at 4 °C in PBS 1× for later validation by immunocytochemistry.

### 2.4. Immunocytochemistry

The expression of pluripotency and three germ layers markers was evaluated by immunocytochemistry. The following primary antibodies were used: anti-OCT4 (1:400, Abcam ab19857, Cambridge, UK), anti-SSEA4 (1:66, Abcam ab16287, Cambridge, UK), anti-TRA-1-60 (1:100, Abcam ab16288, Cambridge, UK), anti-SOX2 (1:1000, Abcam ab97959, Cambridge, UK), anti-NESTIN (1:500, Merck Millipore ABD69, Burlington, MA, USA), anti-β-III TUBULIN (1:200, Merck Millipore MAB163, Burlington, MA, USA), anti-SMA (1:200, Dako M0851, Glostrup, Denmark) and anti-GATA4 (1:500, Santa Cruz Biotechnology, sc-25310, Dallas, TX, USA). Succinctly, cells in µ-Slide 8-well ibiTreat chamber slides (ibidi) were washed twice with PBS 1× and permeabilized with 0.2% Triton X-100 in PBS 1× for 2 min at RT. Non-specific binding epitopes were blocked by incubating cells with PBS 1× 3% BSA for 30 min. Primary antibodies were incubated in a PBS 1× 3% BSA solution overnight at 4 °C. The following day, cells were washed twice with PBS 1× and incubated for 2 h with the secondary antibodies anti-rabbit Alexa Fluor-488 conjugate (Invitrogen A-11008, Waltham, MA, USA) and anti-mouse Alexa Fluor-568 conjugate (Invitrogen A-11031, Waltham, MA, USA), diluted 1:200 in PBS 1× 3% BSA. The nuclei were stained with Hoechst 33342 (1 μg/mL) for 5 min in the dark and cells were again washed twice in PBS 1× and kept in PBS 1× at 4 °C until observation. Images were acquired using a Zeiss Confocal LSM 710 microscope with a 20× or 40× objectives and processed using Zen Black Edition, version 2.3 (Zeiss, Oberkochen, Germany).

### 2.5. PCR and Quantitative Real-Time PCR (qPCR)

Total RNA from hiPSCs and organoids was extracted using the NucleoSpin RNA Isolation Kit (Macherey-Nagel, Düren, Germany), according to the manufacturer’s recommendations for cultured cells. Complementary DNA (cDNA) was synthesized from 400 ng of total extracted RNA using the NZY First-Strand cDNA Synthesis Kit (Nzytech, Lisboa, Portugal), following the instructions from the supplier. qPCR was performed in a StepOnePlus thermocycler (Applied Biosystems, Waltham, MA, USA) using 96-well low-profile microtiter plates (Nerbe Plus, Winsen, Germany) and employing the NZYSpeedy qPCR Green Master Mix 2× (Nzytech, Lisboa, Portugal) for mRNA quantification. Values were normalized to the expression of *HPRT.* PCR primer sequences were the following (gene name: 5′–3′ forward primer/reverse primer):

*OCT4*: CTCCAACATCCTGAACCTCAGC/CTGCTTTGCATATCTCCTGAAG
*SOX2*: GCCGAGTGGAAACTTTTGTCG/GGCAGCGTGTACTTATCCTTCT
*NANOG*: CGTCACACCATTGCTATTCTTG/CTCCAACATCCTGAACCTCAGC
*HPRT*: TGACACTGGCAAAACAATGCA/GGTCCTTTTCACCAGCAAGCT
*NCAM1*: GACATCACCTGCTACTTCCTG/GGCTCCTTGGACTCATCTTTC
*NESTIN*: CTCAGCTTTCAGGACCCCAA/ACAGGTGTCTCAAGGGTAGC
*βIII-TUBULIN:* CGTCCACAGTTCTGGGAAGT/TGTGAGAAGAGGCCTCGTTG

PCR amplification to validate the endogenous expression of pluripotency genes *OCT4* and *SOX2* and the loss of episomal expression from the reprogramming vectors was performed using the MyTaq (Bioline, London, UK) and 0.1 mM forward and reverse primers (described by Sladen et al. [43]) loaded with 200 ng cDNA. PCR reactions were incubated in a thermocycler for a total of 30 cycles before analysis via gel electrophoresis.

### 2.6. Karyotyping

Karyotype processing and analysis of each generated hiPSC cell line was performed by Laboratórios Germano de Sousa (Lisbon, Portugal). Thirty independent metaphases were counted.

### 2.7. Sequencing

DNA was extracted from hiPSCs using the NZY Tissue gDNA Isolation kit (Nzytech, Lisboa, Portugal), following the manufacturer’s instructions. The entire coding region of the *GRN* gene (NM_002087.3) was amplified by PCR using specific primer sequences flanking the intron-exon boundaries (primer sequences are available on request). Genomic DNA (100 ng) was amplified in a 25 μL reaction volume, using 0.5 μM of each primer, 1.5 mM MgCl_2_, 0.2 mM dNTPs and 0.5 unit of Taq polymerase (Promega, Madison, WI, USA). The amplicons were purified using a High Pure PCR Product Purification Kit (Roche, Basel, Switzerland), according to the manufacturer’s protocol. Subsequently, the PCR products were directly sequenced on a capillary automated sequencer CEQ 8000 (Beckman Coulter, Brea, CA, USA). The presence of the pathogenic variant and the co-segregation studies were performed on a separate amplification of the exon 9 of the *GRN* gene with subsequent direct sequencing.

### 2.8. Generation of Human Whole-Brain Organoids

Whole-brain organoids were generated based on the protocol published by Lancaster et al. [42,44], with modifications presented in the STEMdiff Cerebral Organoid Kit (Stem Cell Technologies, Vancouver, Canada). In brief, hiPSC colonies were dissociated using Accutase (Stem Cell Technologies, Vancouver, Canada). A total of 9000 cells per well were plated on a 96-well cell suspension plate (Greiner Bio-One) in EB formation medium, supplemented with 10 μM ROCK inhibitor Y-27632 (Stem Cell Technologies, Vancouver, Canada). Embryoid bodies were fed on day 2 and day 4 with EB formation medium and transferred to 24-well cell suspension plates (Greiner Bio-One, Kremsmünster, Austria) in induction medium, on day 5. On day 7, EBs were embedded on droplets of cold Matrigel (Corning, Corning, NY, USA) on a sheet of Parafilm. The droplets were allowed to polymerize at 37 °C for 30 min and were subsequently removed from the Parafilm and grown in expansion medium for 3 days in ultra-low-attachment 6-well plates (Corning, Corning, NY, USA). Afterwards, the EB droplets were kept in suspension, under rotation (65 rpm), in maturation medium (Stem Cell Technologies, Vancouver, Canada). Whole-brain organoids were maintained in maturation medium until 3 months of differentiation.

### 2.9. Immunohistochemistry

Brain organoids were collected at 2 months of differentiation. Upon collection, they were fixed in PBS 1× 4% PFA for 1 h 30 min and then transferred to a solution of 30% sucrose in PBS 1× for cryoprotection. Subsequently, they were transferred into OCT embedding matrix (VWR), snap-frozen on dry ice and stored at −80 °C. Using a cryostat, 20 μm thick sections were obtained and collected directly into adhesive Superfrost Plus slides (Thermo Scientific Menzel). Cryosections were washed twice with PBS 1× to remove the excess of OCT and permeabilized with 0.5% Triton X-100 in PBS 1× for 15 min at RT. Sections were then blocked in PBS 1× 3% BSA for 1 h at RT and incubated with anti-NESTIN (1:500, Merck Millipore ABD69, Burlington, MA, USA) and anti-MAP2 (1:1000, Synaptic Systems #188004, Goettingen, Germany) primary antibodies diluted in PBS 3% BSA, overnight, at 4 °C. The next day, sections were washed twice with PBS 1× and incubated for 2 h at RT, with the secondary antibodies anti-rabbit Alexa Fluor-488 conjugate (Invitrogen A-11008, Waltham, MA, USA) and anti-guinea pig Alexa Fluor-647 conjugate (1:500, Invitrogen A-21450, Waltham, MA, USA) diluted 1:200 in PBS 1× 3% BSA. Nuclei were stained with Hoechst 33342 (1 μg/mL) for 5 min in the dark. Sections were washed twice in PBS 1× and mounted for microscopy using Dako Fluorescent Mounting Medium (Dako). Images were acquired using a Zeiss Confocal LSM 710 microscope with a 20× objective and processed using Zen Black Edition, version 2.3 (Zeiss, Oberkochen, Germany).

### 2.10. Tissue/Cells Collection for Western Blotting

hiPSC pellets and 3-month-old brain organoids were collected, individually, and stored at −80 °C. Subsequently, samples were homogenized at 4 °C in RIPA lysis buffer (150 mM NaCl, 1% Triton X-100, 0.5% sodium deoxycholate, 0.1% sodium dodecyl sulfate (SDS), 25 mM Tris pH 7.4), supplemented with 10% CLAP, 0.5 mM dithiothreitol (DTT) and 1 mM phenylmethylsulfonyl fluoride (PMSF). Freeze and thaw cycles were complemented with sonication to improve cell membrane disruption. Samples were centrifuged at 21,100× *g* for 20 min at 4 °C. The supernatant was collected, and protein content was determined using the Bio-Rad DC protein assay kit (Bio-Rad, Hercules, CA, USA), following the manufacturer’s instructions. Protein samples (20 μg total protein in hiPSC samples and 30 μg in brain organoid samples) were denatured with 4× Laemmli buffer (Bio-Rad, Hercules, CA, USA) and 10% β-mercaptoethanol and boiled at 95 °C for 5 min. Samples were frozen at −20 °C until further use in Western blot.

### 2.11. Western Blotting

Equal amounts of total protein were run on 8% polyacrylamide gels at 70–90 volts. Proteins were transferred to Immobilon-P PVDF membranes (Merck Millipore, Burlington, MA, USA) for 2 h at 1000 mA and blocked at RT for 1 h in 5% non-fat milk diluted in TBS 1× (25 mM Tris-HCl, 150 mM NaCl) with 0.1% Tween-20 (TBS-T), following primary antibody anti-PGRN (1:1000, Abcam ab208777, Cambridge, UK) incubation at 4 °C overnight. After 3 washes in TBS-T, the membranes were incubated with the appropriate secondary antibody (1:10,000) at RT for 2 h. The membranes were then washed 3 times in TBS-T and incubated with Vistra EFC (Enhanced Chemifluorescence) substrate (Merk, Darmstadt, Germany ) for 5 min at RT. The fluorescence signal was visualized using a ChemiDoc System (Bio-Rad, Hercules, CA, USA) and analysis was performed using the Image Lab Software (Bio-Rad, Hercules, CA, USA). Anti-GAPDH (1:5000, Merck Millipore MAB374, Burlington, MA, USA) was used as a loading control. The secondary antibodies employed were alkaline phosphatase affinipure goat anti-mouse IgG (Jackson Immunoresearch, #115-055-146, West Grove, PA, USA) and alkaline phosphatase affinipure mouse anti-rabbit IgG (Jackson Immunoresearch, #211-055-109, West Grove, PA, USA).

### 2.12. Statistical Analysis

Data are represented as mean values ± s.e.m. (standard error of the mean). Statistical analysis was calculated using one-way ANOVA, following Dunnett’s multiple comparisons test. Analysis was performed using the standard statistical software GraphPad Prism 8. Differences were considered statistically significant for *p* values < 0.05 (* *p* < 0.05, ** *p* < 0.01, *** *p* < 0.001, **** *p* < 0.0001).

## 3. Results

### 3.1. Generation and Characterization of Patient-Derived hiPSCs

**Figure 2:**
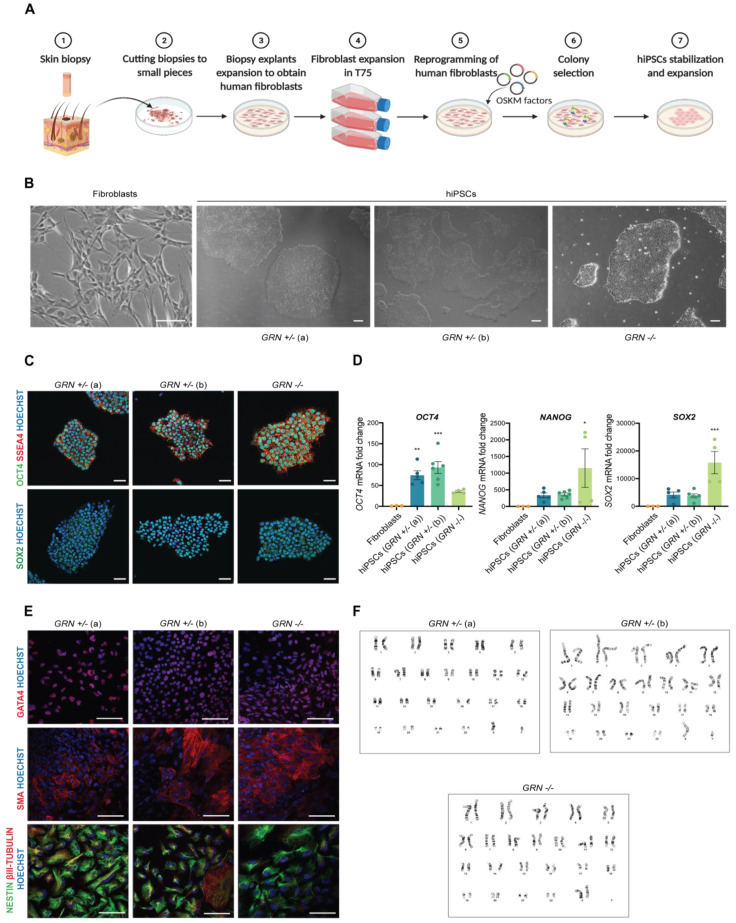
Generation and characterization of patient-derived hiPSCs. hiPSCs were generated from skin fibroblasts of three individuals, from the same family, all carriers of the progranulin mutation c.900_901dupGT. One individual harboring a homozygous mutation and two individuals harboring a heterozygous mutation. (**A**) Schematic representation of the protocol for fibroblast isolation and reprogramming into hiPSCs. (**B**) Typical morphology of fibroblasts and hiPSCs following reprogramming (scale bar: 200 μm). (**C**) Expression of pluripotency markers OCT4, SSEA4 and SOX2 was detected by immunocytochemistry in the three hiPSC lines (scale bar: 50 μm). (**D**) Increased expression of pluripotency genes *OCT4*, *NANOG* and *SOX2,* with respect to fibroblasts, was also observed by qPCR in at least two independent hiPSC colonies from each individual (*n* = 3 fibroblast lines; *n* = 4 *GRN*^+/−^ (a) colonies; *n* = 5 *GRN*^+/−^ (b) colonies; *n* = 2 *GRN*^−/−^ colonies). (**E**) Expression of trilineage markers GATA4 (endoderm), SMA (mesoderm) and NESTIN and βIII-TUBULIN (ectoderm) (scale bar: 100 μm) was determined by immunocytochemistry in the two most promising colonies of each hiPSC lines (described in Table 1), following the three germ layer assay. (**F**) A normal karyotype, with 46 chromosomes *GRN*^+/^^−^ (a): 46, XY, *GRN*^+/−^ (b): 46, XY, *GRN*^−/−^: 46, XX was identified in both colonies of the three hiPSC lines. One-way ANOVA, following Dunnett’s multiple comparisons test. Data are presented as mean ± s.e.m. Statistical significance: * *p* < 0.05, ** *p* < 0.01, *** *p* < 0.001.

In order to study the cellular consequences of the c.900_901dupGT *GRN* mutation in human brain cells, we generated hiPSCs from primary cultures of dermal fibroblasts collected from three individuals, from the same family, which presented either a homozygous (*n* = 1) or heterozygous mutation (*n* = 2) (Figure 2A,B). For this purpose, skin biopsies were obtained from each individual and cultured until giving rise to a stable population of dermal fibroblasts. The fibroblasts were then reprogrammed using an integration-free strategy, based on nucleofection, to overexpress *OCT3/4*, *SOX2*, *KLF4*, *L-MYC* and microRNA (miR) 302/367 cluster. For each patient, several independent hiPSC-like clones were generated and picked to Matrigel-coated plates. After several days of expansion, hiPSCs displayed specific stem cell-like morphology (multiple round-shaped colonies with distinct borders and a high nuclear to cytoplasm ratio) (Figure 2B). Even though cell morphology is an important indicator of hiPSC status, other tests were performed to further validate the quality of the newly generated hiPSC lines. The pluripotency potential of each original hiPSC clone was confirmed by immunocytochemistry and qPCR. All hiPSC clones (four different clones for *GRN^+/−^* (a), five different clones from *GRN^+/−^* (b) and two different clones from *GRN^−/−^*) presented expression of the pluripotency markers OCT4, SSEA4 and SOX2 (Figure 2C), which was not found in fibroblasts (Supplementary Figure S1) and was confirmed to be endogenous and not dependent on the episomal vector employed for reprogramming (Supplementary Figure S2). Additionally, an increase in the expression of the pluripotency genes *OCT4*, *SOX2* and *NANOG,* with respect to the primary cell source (fibroblasts) (Figure 2D), was also detected by qPCR. Nevertheless, we found that the expression levels of these pluripotency markers diverged between different patient samples and, within the same patient, in different clones (Figure 2D).

The most promising hiPSC clones (two from each individual) were cultured in conditions that potentiated the formation of embryoid bodies to evaluate their ability to differentiate spontaneously into each of the three-germ layers. Following 5 days in EB formation medium, EBs were transferred to Matrigel-coated plates and cultured for another 11 days in serum-containing medium. The presence of trilineage markers was determined by immunocytochemistry at the end of this period. GATA4 was used as an endodermal marker, SMA as a mesodermal marker and NESTIN and β-III TUBULIN as ectodermal markers. It was possible to observe the positive expression for markers of the three germ layers (Figure 2E) in all tested clones from the three hiPSC lines. Chromosomal integrity in the newly generated hiPSCs was also examined in the same two most promising hiPSC clones of each individual. Our results showed that all clones presented a normal karyotype: 46, XY (heterozygous carriers of the *GRN* mutation) or 46, XX (homozygous carrier of the *GRN* mutation) (Figure 2F). All the hiPSC clones were further tested for mycoplasma before clone expansion and freezing at low passage number (6–8 passages). General features of the generated cell lines are reported in Table 1.

### 3.2. Patient-Derived hiPSCs Preserve the GRN Genotype

**Figure 3:**
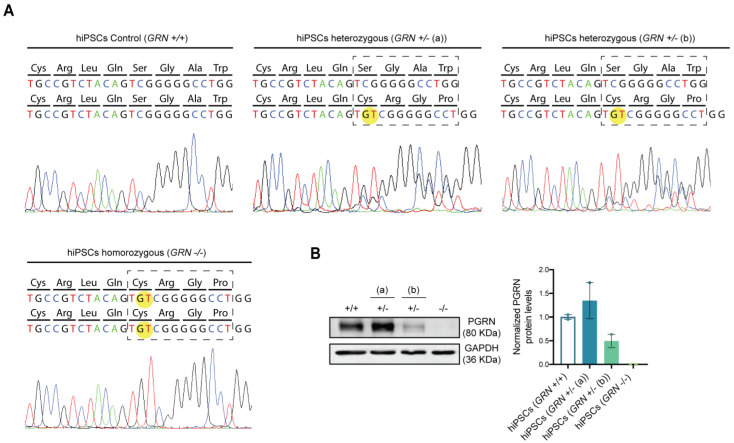
FTLD patient-derived hiPSCs present different progranulin genotypes. After reprogramming, DNA sequencing and protein expression of the newly established hiPSC lines from the three different individuals were assessed and the presence of progranulin mutation c.900_901dupGT was confirmed in all colonies generated from each of the three individuals. (**A**) Electropherogram of the regions containing the studied mutation in the generated hiPSCs shows the GT duplication in one allele from the patient harboring the heterozygous mutation and in both alleles from the patient harboring the homozygous mutation. (**B**) Western blot quantification of PGRN protein levels was performed in the two most promising hiPSC colonies from each individual (described in Table 1). Representative Western blot membrane and quantification of PGRN band density levels, normalized to GAPDH, show a decrease in PGRN protein levels in both disease conditions (*GRN*^+/−^ (b) and *GRN*^−/−^), except for the asymptomatic heterozygous individual (*GRN*^+/−^ (a)), which shows an increase in PGRN protein levels. Data are presented as mean ± s.e.m.

After the broad characterization of the newly generated hiPSC lines, we wanted to analyze whether the genotype of each line suffered alterations due to the reprogramming process. For this purpose, the region within the *GRN* gene bearing the mutation was sequenced in each hiPSC line. As expected, the GT duplication, c.900_901dupGT (p.Ser301Cysfs*61), was identified in a single allele in the hiPSC clones from the individuals bearing the heterozygous mutation and in both alleles in the hiPSC clones from the patient with the homozygous mutation (Figure 3A).

It has previously been described that *GRN* loss of function mutations lead to a reduction in serum progranulin levels that reaches more than a 50% loss in symptomatic FTLD patients, while NCL patients with homozygous mutations present a complete absence of the protein [18]. To assess if the generated hiPSC lines complied with the expected phenotype, PGRN expression levels were evaluated by Western blot (Figure 3B). We observed a significant reduction (approximately 50%) in PGRN protein levels in hiPSC clones from one of the heterozygous patients (*GRN^+/−^* (b)), while no PGRN protein could be detected in hiPSC clones from the homozygous patient (100% reduction). Interestingly, hiPSC clones from one of the heterozygous individuals (*GRN^+/−^* (a)) presented an increase in PGRN levels (Figure 3B) with respect to control lines, which may be related to the fact that this individual was young and still asymptomatic at the time of the biopsy collection.

### 3.3. Patient-Derived hiPSCs Can Generate Brain Organoids

**Figure 4:**
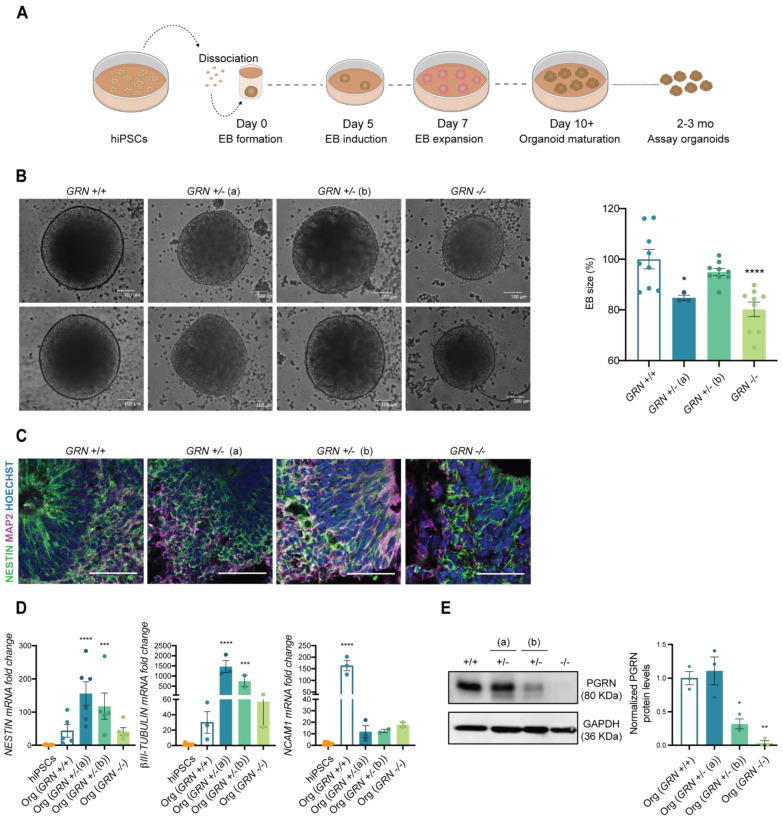
FTLD patient-derived hiPSCs are capable of generating brain organoids. One colony of each newly established hiPSC lines was used to generate human 3D brain models (NCBL1.c5; NCBL2.c46; NCBL3.c6 and NCBL4.c27). (**A**) Schematic representation of the organoid generation, following the protocol established by Lancaster and colleagues. (**B**) EBs size was determined at day 5 of the protocol and is presented as a % of control genotype (*n* = at least 3 organoids per genotype). EBs from *GRN*^+/^^−^ (a) and *GRN*^−/^^−^ genotypes show a decrease in mean size. (**C**) The neuronal precursor NESTIN and neuronal marker MAP2 are expressed in 2-month-old generated brain organoids, as observed by immunohistochemistry. (**D**) Increased expression of ectodermal genes (*NESTIN*, *βIII-TUBULIN*, *NCAM1*) was also observed in the generated brain organoids with respect to hiPSCs (*n* = at least 3 organoids per genotype). (**E**) Representative Western blot membrane and quantification of PGRN in 3-month-old total extracts of whole-brain organoids (*n* = 3 organoids per genotype), normalized to GAPDH, shows a reduction in protein levels of PGRN in brain organoids of both *GRN*^+/^^−^ (b) and *GRN*^−/^^−^ conditions, while the *GRN*^+/^^−^ (a) condition showed similar levels to control. One-way ANOVA following Dunnett’s multiple comparisons test. Data are presented as mean ± s.e.m. Statistical significance: * *p* < 0.05, ** *p* < 0.01, *** *p* < 0.001, **** *p* < 0.0001.

Next, we wanted to understand the ability of the developed hiPSC lines to generate specific cell types from the ectodermal lineage, including neurons, in a complex 3D culture system. For this purpose, we employed the protocol established by Lancaster and colleagues [42,44] to originate whole-brain organoids, as demonstrated in Figure 4A. This unguided protocol allows for generating a diversity of cell types that are present in the brain during early embryonic development, and which are of ectoderm origin. In the first step of this process, EBs were formed from one stable hiPSC clone from each individual (at a cell passage above 20) and their size was monitored throughout the first 5 days. Interestingly, at day 5, we could observe a significant decrease in the diameter of the EBs obtained from the *GRN^−/−^* line, as well as in the *GRN^+/−^* (a) line with respect to organoids obtained from control lines (Figure 4B). Nevertheless, we found that all three tested hiPSC lines could generate human brain organoids. At two months in culture, and similarly to controls, organoids from all three new lines presented ventricle-like structures that mimic the initial stages of cortical plate development with expression of the neuronal precursor marker NESTIN (Figure 4C). It was also apparent the presence of the neuronal marker MAP2, more evident in the outer part of the ventricle, reflecting the inside-out neuronal migration (Figure 4C). These findings were complemented by qPCR analysis, which revealed an increase in the expression of ectodermal genes (*NCAM1*, *NESTIN* and *β-III TUBULIN*) at this same age (Figure 4D).

PRGN levels were also analyzed in 3-month-old whole-brain organoids, and the profile of protein expression in total organoid extracts was similar to what was previously observed in hiPSCs (Figure 4E). We found a modest increase in PGRN in brain organoids generated from *GRN^+/−^* (a), a decrease of approximately 60% in brain organoids generated from the *GRN^+/−^* (b) hiPSC line and total loss of PGRN expression in brain organoids generated from the *GRN^−/−^* hiPSC line (Figure 4E).

## 4. Discussion

*GRN* mutations cause a range of neurological disorders, presenting an allele dose-dependent pattern. While homozygous loss-of-function *GRN* mutations are very rare and result in a specific subtype of neuronal ceroid lipofuscinosis (CLN11), heterozygous mutations in this gene are more common and, in the case of the Portuguese population, constitute the second leading cause of genetic FTLD, a rapidly progressing type of dementia. Interestingly, while most neurodegeneration-related risk genes, such as *APP* and SOD1, are usually associated with a specific disease, *GRN* mutations and polymorphisms appear to be involved in a diverse group of neurological conditions, ranging from FTLD to ALS, AD, PD and even autism. This places the *GRN* gene in a unique position to regulate brain health.

Recent advances in our understanding of the cellular and molecular consequences of reduced PGRN levels have established a crucial role for this protein in lysosomal health [12], neuronal survival [1,2], synaptic pruning and neuroinflammation [7,8,9,10]. Despite these insights, PGRN mechanisms of action and main signaling cascades are still poorly understood. For example, although we know that PGRN is cleaved into a set of smaller proteins named granulins inside the lysosome, little is known concerning the interplay of PGRN with its cleavage products and the role of granulins in brain homeostasis. Other relevant questions that remain unanswered include: (1) How does PGRN impact autophagy? (2) How do PGRN levels change throughout life, and what epigenetic mechanisms regulate PGRN expression? (3) Is there a critical period during development when PGRN is essential? (4) Is restoring PGRN expression a therapeutic avenue to treat neurodegeneration and, if so, (5) how tightly do we need to control PGRN levels given its trophic properties?

In order to answer these questions, we believe that it is crucial to develop novel humanized research models that allow to simultaneously preserve the genetic background of individuals bearing *GRN* mutations and recapitulate the major pathophysiological hallmarks observed in FTLD and other *GRN*-associated diseases. With this in mind, we generated hiPSCs from dermal fibroblasts obtained from several members of a Portuguese family where cases of both FTLD-*GRN* and CLN11 had been previously diagnosed [18,19].

Ever since Yamanaka et al. reported that overexpressing OCT4, SOX2, KLF4 and MYC (OSKM) in mouse fibroblasts would induce the recapitulation of embryonic stem cell features and abilities [45], an important ethical barrier (experimenting with human embryos) was overcome. The emerging realm of induced pluripotent stem cells has revolutionized the ability of scientists to produce better and cheaper models of human system homeostasis and human diseases. Although the discovery of OSKM factors was a landmark for iPSC development, it was just the first step in this blooming field of science. Since then, several other reprogramming factors were identified and described to improve reprograming of different types of human and rodent cells, including fibroblasts and blood cells [46,47]. In this work, human fibroblasts were effectively reprogramed by episomal nucleofection of the four Yamanaka factors: OCT3/4, SOX2, KLF4 and MYC, together with the miR 302/367 cluster [48,49]. To ensure that the newly generated cell lines were indeed iPSCs, quality control methods were used for their characterization, according to the currently accepted best practices for the characterization of iPSCs [50], including cell morphology analysis, expression of pluripotency-associated markers, ability to differentiate into the three germ layers and karyotype analysis.

Since iPSCs should be able to originate any cell type, they need to exhibit both pluripotency and the ability to differentiate into a diverse range of cells, with origins in different embryonic leaflets. In this study, both criteria were met for the cells from all PGRN mutation carriers (Figure 2C–E). However, different levels of endogenous expression of pluripotency genes were observed, which may be related to the different genetic backgrounds of the patients or due to the differences in reprogramming efficiency of each fibroblast line. Nevertheless, loss of episomal-driven expression of the pluripotency genes was confirmed by PCR for the most promising clones of each hiPSC line, by using primers designed against the endogenous or vector-encoded pluripotency genes [43] (Supplementary Figure S2).

Currently, the trilineage assay is considered the standard method to evaluate iPSCs’ ability to generate EBs and spontaneously differentiate into the three germ layers: endoderm, mesoderm and ectoderm (Figure 2E). This assay has replaced the previously preferred teratoma assay, which consisted on the injection of iPSCs into an immune-deficient mice to assess the ability of these cells to generate a tumor [26,51]. By replacing the teratoma assay by the trilineage assay, the same conclusion can be drawn from a less expensive and time-consuming method, while also avoiding the use of animal models. All three cell lines developed in this work were able to form EBs and express the three lineage markers SMA, GATA4, NESTIN and βIII-TUBULIN, which confirmed their iPSC status.

Since the reprogramming process is often associated with high mutation rates, which may lead to the accumulation of genomic abnormalities, karyotyping analysis is also a standard procedure in iPSC characterization. All the generated lines maintained the normal karyotype (Figure 2F) at passage 20. However, genomic abnormalities can accumulate during long-term culturing [52], becoming essential to repeat the analysis over time or restrict use to low cell passages. Additionally, it may be useful to employ other methods to detect genomic alterations not reported by karyotyping, including single nucleotide polymorphisms (SNPs) and copy number variation (CNV) arrays [53,54].

Considering the strong impact of *GRN* mutations in the brain, an organ that originates from the ectoderm germ layer, we decided to further test the ability of these new lines to generate ectoderm-specific cells through the formation of 3D brain organoids (Figure 4B–D). Upon neuronal induction, we confirmed the expected increase in several ectodermal (NESTIN, βIII-TUBULIN and NCAM1) and neuronal markers (NESTIN and MAP2) (Figure 4C,D) [42], as compared to the respective hiPSC line. Within the same genotype, some degree of variability was detected in the expression of these genes, which may be related to the intrinsic variability associated with the unguided organoid protocol [44].

Lastly, and to evaluate whether the generated cell lines presented the expected reduction in PGRN, typically observed in FTLD patients with *GRN* mutations, PGRN levels were assessed by Western blot in both hiPSCs (Figure 3B) and brain organoids (Figure 4E). The profile of PGRN expression was similar in hiPSCs and organoids, and recapitulated progranulin levels in primary fibroblasts and blood cells from the carriers (data not shown), showing that the genetic feature in study is efficiently preserved throughout the reprograming and differentiation protocols. As expected, we observed a complete loss of PGRN expression in the homozygous mutation carrier (*GRN^−/−^*), and a significant reduction in one of the heterozygous carriers *GRN^+/−^* (b). Interestingly, the other heterozygous carrier (*GRN^+/−^* (a)) presented levels of PGRN expression similar to those of the control. Since this individual was relatively young and still asymptomatic at the time of the skin biopsy, these results may reflect a case of haplosuficiency that can later evolve to haploinsufficiency, as the organism ages and loses the ability to produce the necessary protein to preserve normal function from a single gene copy. In addition, it is important to note that no truncated protein was detected by Western blot in any of the individuals bearing the mutation, probably since this particular mutation causes nonsense-mediated mRNA decay that leads to the quick degradation of the mutated transcript prior to protein production. Taken together, these observations corroborate the hypothesis that PGRN levels change throughout life and tend to decrease with aging, hinting at a possible compensatory mechanism that sustains high levels of PGRN expression in *GRN* mutation carriers and that, once lost, contributes to the onset of FTLD symptoms.

## 5. Conclusions

In this study, patient-derived human dermal fibroblasts bearing *GRN* mutations were successfully reprogrammed into hiPSCs. The newly generated hiPSCs can be further differentiated into cell types of the three germ layers, including neurons, as well as employed to generate complex 3D cellular models, such as brain organoids, with the ability to recapitulate the genetic and molecular features of the original patients’ cells. Based on these findings, we believe these hiPSC lines constitute a set of novel research tools which may prove relevant to further our understanding of the cellular and molecular mechanisms associated with FTLD and CLN11, and for the identification and testing of novel therapeutic approaches.

## Figures and Tables

**Table 1:** General features of the newly generated cell lines.

Unique Stem Cell Line Identifier	NCBL1.c5	NCBL2.c11; NCBL2.c46	NCBL3.c2; NCBL3.c6	NCBL4.c27; NCBL4.c33
Alternative name of stem cell line	*GRN^+/+^*	*GRN^+/−^* (a)	*GRN^+/−^* (b)	*GRN^−/−^*
Institution	Center for Neuroscience and Cell Biology, University of Coimbra (CNC, UC)			
Contact information of distributor	Ana Luísa Cardoso: uc41483@uc.pt			
Type of cell line	Induced pluripotent stem cells (iPSCs)			
Origin	Dermal human fibroblasts			
Individual age and sex	36 years old; male	29 years old; male	63 years old; male	34 years old; female
Method of reprogramming	Episomal nucleofection: OSKM factors (OCT3/4, SOX2, KLF4 and MYC, together with the miR 302/367 cluster)			
Genetic modification	No	Yes	Yes	Yes
Associated disease	-	Frontotemporal lobar degeneration (FTLD)	Frontotemporal lobar degeneration (FTLD)	Neuronal ceroid lipofuscinosis (NCL11)
Gene/locus	-	Granulin (*GRN*), 17q21.31		
Date archived/stock date	2021			
Cell line repository/bank	RRID:CVCL_C0P1	RRID:CVCL_C0P2; RRID:CVCL_C0P3	RRID:CVCL_C0P4; RRID:CVCL_C0P5	RRID:CVCL_C0P6; RRID:CVCL_C0P7
Ethical approval	The study was approved by the Ethics Committee of the Faculty of Medicine, University of Coimbra (Project CE-028/2016)			

## Data Availability

The data presented in this study are openly available in [ExPASy—Cellosaurus: RRID:CVCL_C0P1; RRID:CVCL_C0P2; RRID:CVCL_C0P3; RRID:CVCL_C0P4; RRID:CVCL_C0P5; RRID:CVCL_C0P6; RRID:CVCL_C0P7.

## Supplementary Materials

The following supporting information can be downloaded at: https://www.mdpi.com/article/10.3390/biomedicines10081905/s1, Figure S1: Pluripotency markers OCT4, SSEA4 and SOX2 are expressed in hiPSCs, but not in skin fibroblasts, Figure S2: Representative gel electrophoresis of the PCR amplification products obtained from the CDS or plasmid transcripts of SOX2 and OCT4.
