# Supplementary material for: Generation and Characterization of Novel iPSC Lines from a Portuguese Family Bearing Heterozygous and Homozygous GRN Mutations
Source: Biomedicines. 2022 Aug 6;10(8):1905. doi: 10.3390/biomedicines10081905 (PMC9405606; doi:10.3390/biomedicines10081905)
Supplement: Supplementary file 1 [file biomedicines-10-01905-s001.zip › biomedicines-1828824-supplementary.pdf]

Supplementary Figure S1

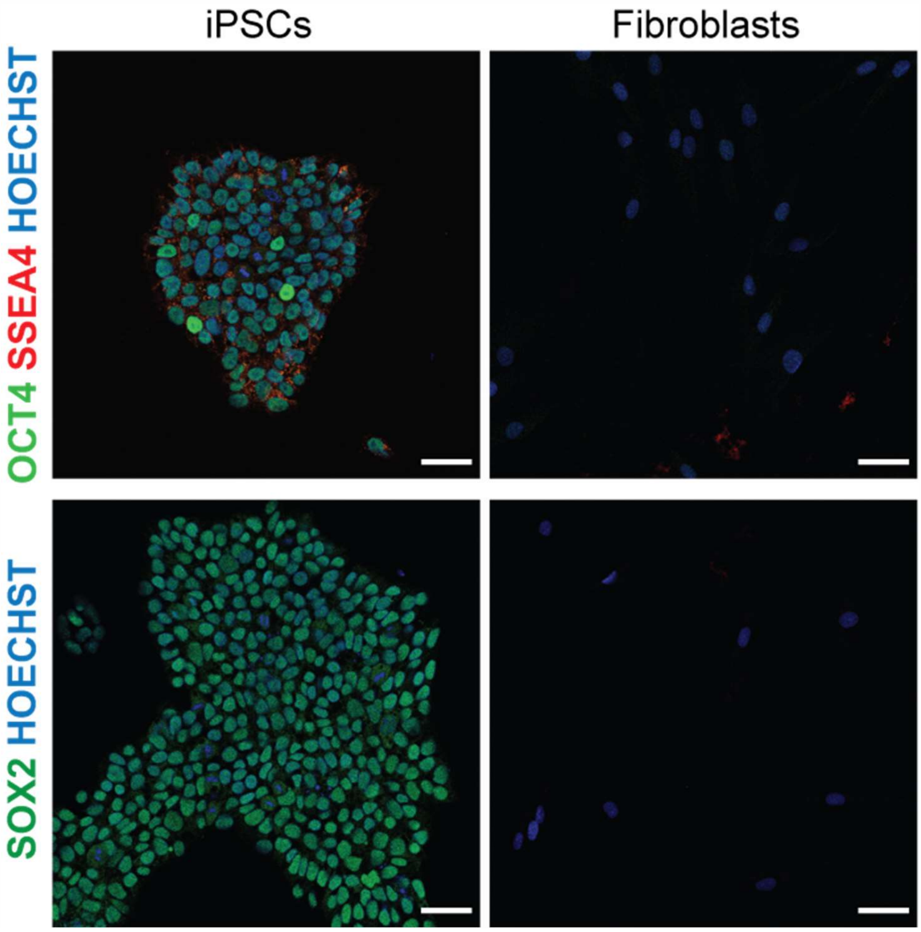

Supplementary Figure S1 – Pluripotency markers OCT4, SSEA4 and SOX2 are expressed in hiPSCs, but not in skin fibroblasts. Expression of pluripotency markers OCT4, SSEA4 and SOX2 was detected by immunocytochemistry in the NCBL1.c5 hiPSC line but not in fibroblasts from the same donor (scale bar: 50 μm).

Supplementary Figure S2

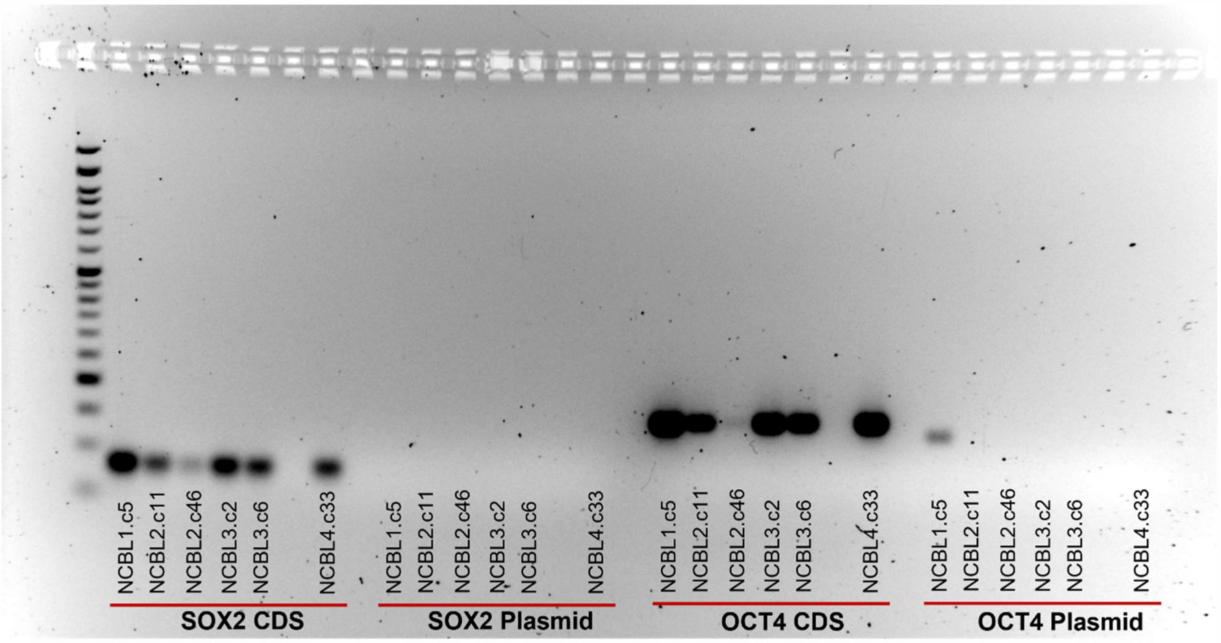

Supplementary Figure S2 – Representative gel electrophoresis of the PCR amplification products obtained from the CDS or plasmid transcripts of *SOX2* and *OCT4*. RNA was extracted and transcribed from each new hiPSC line, between passages 16 and 20. The cDNA was amplified employing primers specific for the endogenous transcript (CDS) or the transcript derived from the reprogramming vector (Plasmid) for both *SOX2* and *OCT4* genes and run in a electrophoresis gel. All tested hiPSCs clones presented endogenous expression of both *SOX2* and *OCT4* and none or very small expression of the plasmid-derived transcripts
